# Nanoscale morphological and structural analysis of round and donut oligomers formed by C-terminal domain of TDP-43

**DOI:** 10.1039/d6cp01760f

**Published:** 2026-07-02

**Authors:** Davis Pickett, Yana Purvinsh, Joshua T. Skrehot, Daniel Warren, Dmitry Kurouski

**Affiliations:** a Department of Biochemistry and Biophysics, Texas A&M University College Station Texas 77843 USA dkurouski@tamu.edu +979-458-3778 +979-458-3778; b Department of Chemistry, Texas A&M University College Station Texas 77843 USA

## Abstract

Amyotrophic lateral sclerosis (ALS), frontotemporal dementia (FTD), Alzheimer's disease (AD), limbic predominant age-related TDP-43 encephalopathy (LATE), and Parkinson's disease are associated with an abrupt aggregation of TAR DNA-binding protein 43 (TDP-43). Although molecular mechanisms of this pathological aggregation remain unclear, accumulated evidence suggests that the C-terminus domain (C-terminal domain (CTD)) is the trigger of TDP-43 self-assembly into toxic oligomers and fibrils. While the secondary structure and morphology of protein fibrils have been well documented, very little is known about TDP-43 oligomers. This is primarily because of the transient nature and low concentrations of these protein species. In the current study, we utilize nano-infrared spectroscopy, also known as atomic force microscopy-infrared (AFM-IR) spectroscopy, to investigate the morphology and secondary structure of CTD of TDP-43 oligomers formed at the early and middle stages of protein aggregation. This innovative technique allows us to resolve both morphology and secondary structure of individual protein aggregates. We found that at the early stage of protein aggregation, CTD of TDP-43 formed two morphologically different protein aggregates: donut-like (DO) and round (RO) oligomers. DO yielded fibrillar species, while RO persisted throughout the entire course of CTD TDP-43 self-assembly.

## Abstract 2

Amyotrophic lateral sclerosis (ALS), frontotemporal dementia (FTD), Alzheimer's disease (AD), and Parkinson's disease are associated with an abrupt aggregation of TAR DNA-binding protein 43 (TDP-43).

## Graphical abstract

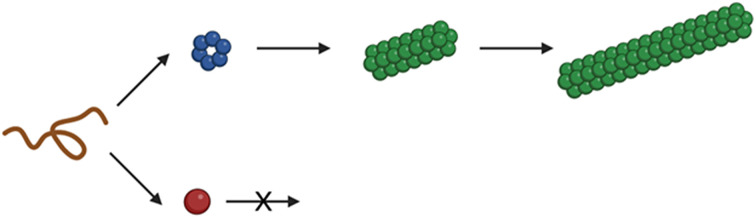

In a healthy state, TDP-43 is a key gatekeeper of the nucleus, where it orchestrates transcription and RNA splicing. However, under pathological conditions, TDP-43 abandons the nucleus, translocating to the cytosol where it sequesters into “stress granules” alongside a chaotic mix of proteins and RNAs.^1–3^ This pathological process is linked to amyotrophic lateral sclerosis (ALS), frontotemporal dementia (FTD), Alzheimer's disease (AD), and Parkinson's disease (PD).^4–9^ The primary driver of protein sequestering and self-assembly is its C-terminal domain (CTD), the sequence enriched with glycine, glutamine, and asparagine residues.^10–14^

*In vitro* studies demonstrated that CTD of TDP-43 spontaneously self-assembles into soluble oligomers that later propagate into β-sheet rich fibrils.^15–18^ While the biophysical properties of CTD of TDP-43 fibrils are well understood, very little is known about protein oligomers. To a large extent, this is because these species are present at very low concentrations during protein aggregation. Furthermore, their transient nature limits the use of cryo-electron microscopy and solid-state nuclear magnetic resonance for their structural characterization. At the same time, CTD of TDP-43 oligomers disrupt nucleocytoplasmic transport and damage nuclear pore complexes.^19,20^ These aggregates also propagate from cell-to-cell by autophagy, which results in the spread of neurodegeneration across different areas of the brain.^21,22^

Experimental findings reported by our group demonstrate that morphology and secondary structure of protein oligomers can be resolved using nano-infrared spectroscopy, also known as atomic force microscopy-infrared (AFM-IR) spectroscopy.^23–27^ In AFM-IR, a gold-coated scanning probe is positioned at the surface of individual protein aggregates.^28–31^ Next, pulsed infrared light is used to trigger thermal expansions in the protein species. Thermal expansions are then reordered by the probe and converted into IR spectra. The amide I band in such spectra can be used to examine the secondary structure of protein aggregates.^32–34^ Using AFM-IR, we recently demonstrated that protein aggregation could yield structurally and morphologically diverse protein aggregates (α-synuclein and amyloid β)^31,35^ or two distinctly different types of oligomers (human islet amyloid precursor protein, hIAPP).^36^ In the former case, structural evolution of antiparallel-to-parallel β-sheet has been observed. In the latter case, protein simultaneously formed donut-like (DO) and round (RO) oligomers that had drastically different secondary structures.^36^ It has also been shown that RO persisted throughout the entire course of protein aggregation, while DO quickly disappeared with fibrillar species appearing. Based on these findings, Warren and co-workers made a conclusion that DO were “on-path” oligomers that yielded fibrils, while RO were “off-path” species that generated no higher-order supramolecular ensembles.^36^

In the current study, we utilize innovative nano-Infrared spectroscopy to investigate the morphology and secondary structure of CTD of TDP-43 oligomers formed at the early, middle, and late stages of protein aggregation. For this, sample aliquots were taken during the onset (20 min), during the middle (2 h), and at the end (4.5 h) of protein aggregation (Fig. S1). Next, atomic force microscopy (AFM) was used to examine the topology of protein aggregates observed at all four time points (Fig. 1). During the onset of protein aggregation (20 min), we observed both DO and RO present. We also observed the same protein species during the middle of CTD of TDP-43 aggregation with approximately the same relative ratio. However, at the late stage (4.5 h), only a very small amount of DO was observed (Fig. S2) with the presence of fibrils and RO, Fig. 1. The height of all of these species varied between 5–12 nm, Fig. 1. It should be noted that AFM images only mean to visualize species present at different time points of protein aggregation and not aim to quantify relative abundance of both DO and RO.

**Fig. 1:**
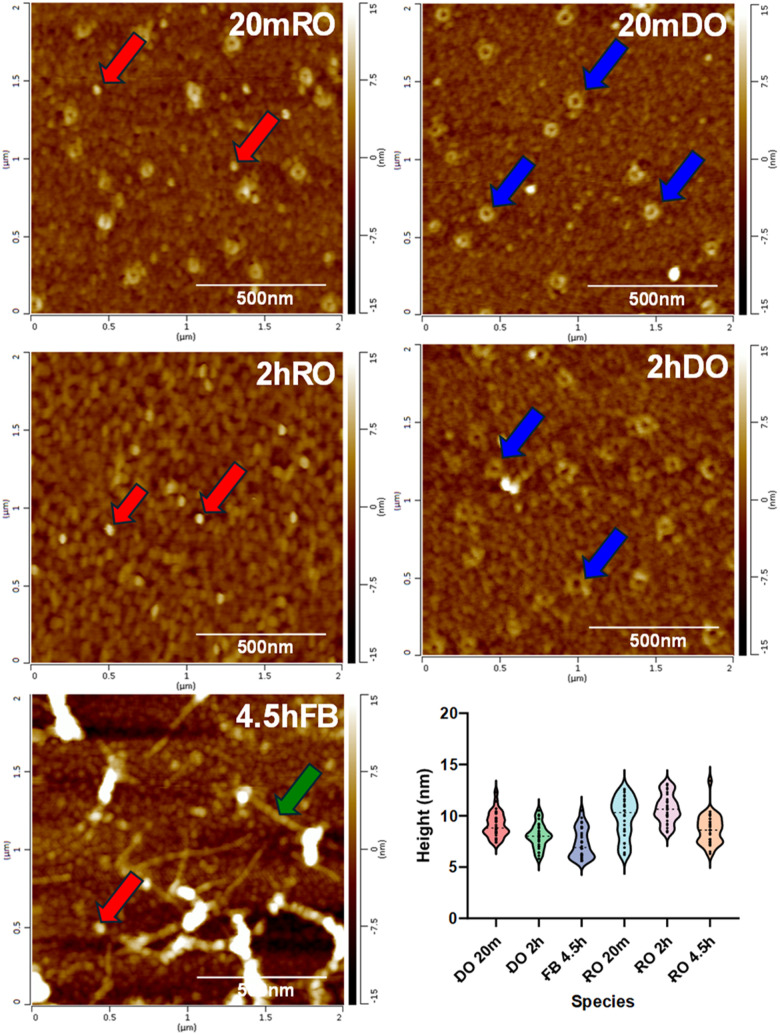
AFM images and a height histogram of protein aggregates observed during the early (20 min), middle (2 h), and at the end (4.5 h) of CTD of TDP-43 aggregation. RO are shown by red, DO by blue, and fibrils by green arrows. Scale bars are 500 nm.

Next, we acquired nano-IR spectra from all protein aggregates discussed above. The spectra exhibited both amide I (1615–1700 cm^−1^) and II (∼1550 cm^−1^) bands. Amide I bonds primarily originate from the C=O vibration of the peptide (amide) bond and, therefore, can be used to interpret the secondary structure of protein aggregates.^37–40^ Expanding upon this, we fit the amide I band in the acquired spectra to reveal the relative contributions of parallel β-sheet, disordered protein, and antiparallel β-sheet secondary structure.

We found that DO that were observed during the early (20 min) and middle (2 h) time points of protein aggregation were dominated by antiparallel β-sheet (∼50%). However, RO observed at the same time points had either equal amounts of parallel β-sheet, disordered protein/α-helix, and antiparallel β-sheet (20 min), or were dominated by disordered protein/α-helix (2 h) with a substantially lower amount of antiparallel β-sheet than corresponding DO. It should be noted that fibrils and RO observed at the end (4.5 h) of CTD of TDP-43 aggregation also had nearly equal amounts of parallel β-sheet, disordered protein/α-helix, and antiparallel β-sheet. Thus, these findings indicate that CTD of TDP-43 forms structurally different oligomers at the early and middle stages of protein aggregation. Although CTD of TDP-43 fibrils observed at the late stages of protein aggregation do not strictly follow the secondary structure of DO (predominance of antiparallel β-sheet), collective analysis of morphology and secondary structure of protein species observed at the early, middle, and late stages of protein aggregation suggests that DO, rather than RO, produced fibrils (Fig. 2).

**Fig. 2:**
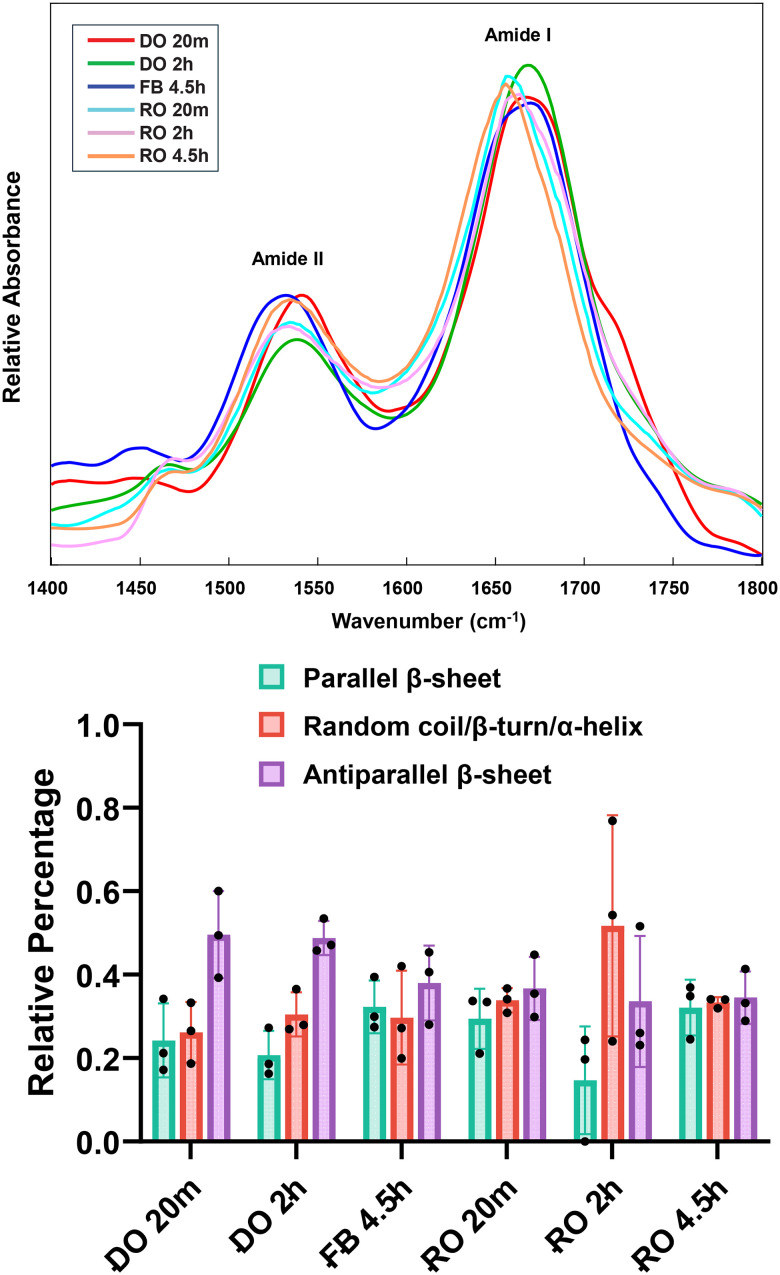
AFM-IR (top) spectra acquired from CTD of TDP-43 aggregates observed during the early (20 min), middle (2 h), and at the end (4.5 h) of protein aggregation. Histogram (bottom) of amide I fit indicating the relative contributions of parallel β-sheet, disordered protein/α-helix, and antiparallel β-sheet in the secondary structure of CTD of TDP-43 aggregates.

Similarly to our findings, previously reported results by Warren and co-workers demonstrated that RO and DO oligomers formed by hIAPP exhibited drastically different IR spectra, which allowed them to resolve differences in their secondary structure.^36^ It should be noted that Warren and co-workers also concluded that DO observed upon hIAPP aggregation seeded fibrils.^36^ Based on current findings on CTD of TDP-43, we can conclude that DO “on-path” and RO “off-path” protein aggregation can be rather a common phenomenon typical for a large group of amyloidogenic proteins rather than a unique case of hIAPP. Certainly, analysis of aggregation dynamics of other amyloidogenic proteins like Tau isoforms, insulin and lysozyme is required to unambiguously make such conclusions.

Summarizing, our results show that during the initial phases of CTD of TDP-43 aggregation, two distinct morphological species emerge: DOs and ROs, Fig. 3. As the process advances, DO oligomers yield fibrillar structures, whereas RO oligomers persist throughout different stages of protein aggregation.

**Fig. 3:**
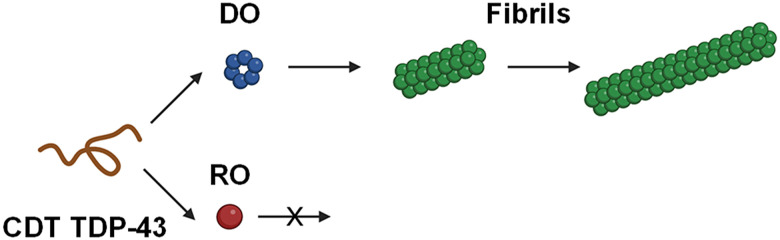
Schematic illustration of “on-path” (blue) and “off-path” (red) aggregation of monomeric CTD of TDP-43 CTD of TDP-43.

## Limitations of the current study

It should be noted that AFM-IR measurements were performed on dried, surface-deposited samples. Furthermore, the study used an isolated CTD fragment aggregated under *in vitro* agitation conditions. Therefore, these experiments may not directly reflect the solution ensemble or the behavior of full-length TDP-43 in cells.

## Experimental section

### Materials

*Escherichia coli* BL21 (DE3) competent cells were purchased from New England BioLabs (C2527H). Isopropyl-ß-d-thiogalactopyranoside (IPTG) was ordered from Fisher Scientific (BP1755-100). Urea, imidazole and dithiothreitol (DTT) were purchased from Sigma-Aldrich (U5378, 288–32-4 and 3483-12-3). β-Mercaptoethanol (BME) and l-histidine were ordered from Thermo Fisher Scientific (21985023 and A10413.22). Ni-NTA Agarose was purchased from Invitrogen (R901-15). Molecular-weight cutoff of 3 kDa was purchased from Sigma-Aldrich (UFC9003).

### TDP-43 expression and purification

We transformed *E. coli* BL21 (DE3) (NEB) with a plasmid carrying the 6xHis-TDP-43 279-360 CTD sequence. Following transformation, we inoculated 50 mL of kanamycin-containing LB medium (25 mg mL^−1^) with a single colony and incubated it overnight (16 h) at 37 °C and 200 rpm. We then scaled the culture to 2 L using the preculture. Upon reaching an OD600 of 0.6, we induced protein production by adding 1 mM IPTG and continued the incubation at 16 °C for an additional 14 hours.

Cells from a 2 L culture were harvested *via* centrifugation at 3000*g* for 15 min at 4 °C. The resulting pellets were resuspended in 80 mL of ice-cold denaturing lysis buffer (6 M urea, 50 mM sodium phosphate pH 8.0, 500 mM NaCl, and 1 mM DTT). Following mechanical lysis using a Microfluidizer LM10, the lysate was clarified by centrifugation at 15 000*g* for 45 min at 4 °C. The supernatant was filtered (0.45 µm) and loaded onto a gravity-flow column containing Ni-NTA agarose beads pre-equilibrated in lysis buffer supplemented with 10 mM imidazole. After washing with 50 mM imidazole, the 6xHis-CTD TDP-43 protein was eluted in 20 mL of buffer containing 300 mM imidazole. Eluted fractions were pooled, analyzed by SDS-PAGE, and concentrated using a 3 kDa MWCO membrane. The final product was dialyzed against 2 × 1 L of buffer (30 mM sodium phosphate pH 8.0, 300 mM NaCl, 2 g L^−1^ histidine). Final protein concentration was determined by UV-vis spectroscopy at 280 nm.

### CTD TDP-43 aggregation

10 µM CTD TDP-43 in a dialysis buffer consisting of 30 mM sodium phosphate (pH 8.0), 300 mM NaCl, and 2 g L^−1^ histidine was transferred to a 96-well plate and incubated at 37 °C for 48 hours with continuous agitation at 510 rpm in a plate reader (Tecan, Männedorf, Switzerland). Protein aggregation kinetics were monitored using a thioflavin T (ThT) fluorescence assay. For this, samples were prepared by mixing with a 25 µM ThT solution and transferring them into a 96-well plate. The plate was incubated in a Tecan plate reader (Männedorf, Switzerland) at 37 °C for 120 hours with continuous agitation at 510 rpm. Fluorescence readings were collected every minute, with an excitation wavelength of 450 nm and an emission wavelength of 488 nm.

### Atomic force microscopy (AFM) imaging and atomic force microscopy-infrared (AFM-IR) spectroscopy

Protein samples (3–6 µL) were deposited onto 70 nm gold-coated silicon wafers and air-dried for 15–20 min. The substrates were subsequently rinsed with deionized water and dried under a gentle stream of dry nitrogen gas. AFM-IR characterization was performed using a NanoIR3 system (Bruker, Santa Barbara, CA, USA) equipped with a QCL laser and ContGB-G contact-mode probes (NanoAndMore). The system was calibrated using a polymethyl methacrylate (PMMA) standard across the 1400–1800 cm^−1^ range. Imaging was conducted at scan rates of 0.5–0.8 Hz with dimensions varying from 5 to 10 µm and a resolution of 512 × 512 pixels. IR spectra were acquired at a resolution of 2 cm^−1^; for each sample, 30 spectra were averaged, with each individual spectrum consisting of three co-averaged acquisitions. Data processing was performed in MATLAB and included Savitzky–Golay smoothing (1-order polynomial, 9 pt window) and mean normalization. Baseline correction and peak fitting were finalized using GRAMS/AI Suite. The spectra were baselined before peak fitting and areas were assigned to each generated peak within the Amide I region. We assigned the following peak windows to respective secondary structures: parallel β-sheet (1610–1640 cm^−1^), random coil (1640–1655 cm^−1^), α-helix/β-turn (1655–1675 cm^−1^), and antiparallel β-sheet (1675–1700 cm^−1^).

## Conflicts of interest

The authors declare no competing financial interests.

## Supplementary Material

CP-028-D6CP01760F-s001

## Data Availability

Data for this paper, including AFM-IR spectra and AFM images are available at repository name at https://zenodo.org/communities/therealkurouskilab/records?q=&l=list&p=1&s=10&sort=newes. Supplementary information (SI): thioflavin T kinetics of protein aggregation. Methods of protein expression, purification, and aggregation, as well as AFM-IR analysis. See DOI: https://doi.org/10.1039/d6cp01760f.
