# Supplementary material for: Nanoscale morphological and structural analysis of round and donut oligomers formed by C-terminal domain of TDP-43
Source: Phys Chem Chem Phys. 2026 Jul 2;28(27):16459–63. doi: 10.1039/d6cp01760f (PMC13325187; doi:10.1039/d6cp01760f)
Supplement: CP-028-D6CP01760F-s001 [file CP-028-D6CP01760F-s001.pdf]

Supplementary Information (SI) for Physical Chemistry Chemical Physics.
This journal is © the Owner Societies 2026

Nanoscale Morphological and Structural Analysis of Round and Donut Oligomers Formed by C-Terminal Domain of TDP-43

Davis Pickett,[1] Yana Purvinsh,[1] Joshua T. Skrehot,[1] Daniel Warren,[1] and Dmitry Kurouski*[1,2]

1. Department of Biochemistry and Biophysics, Texas A&M University, College Station, Texas 77843, United States
2. Department of Chemistry, Texas A&M University, College Station, Texas, 77843, United States

Email: dkurouski@tamu.edu

Supporting Information

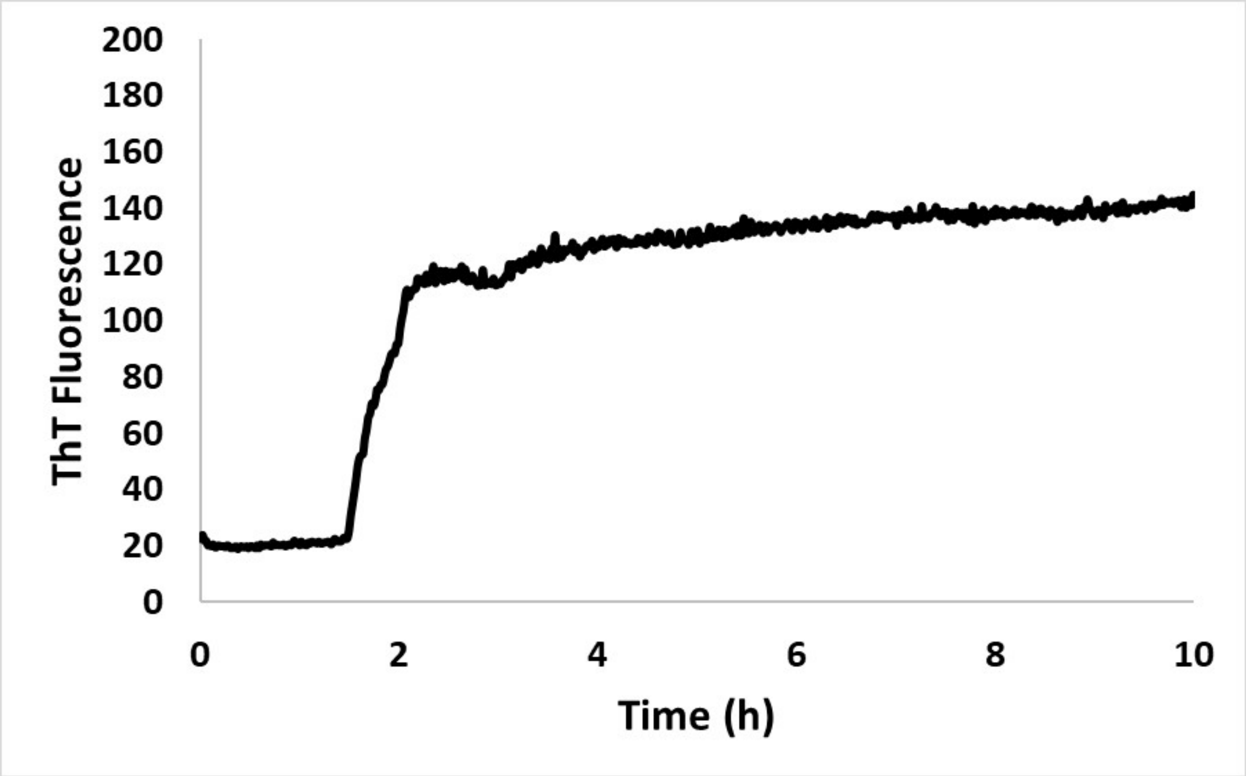

Figure S1: ThT kinetics of TDP-43 C-terminal domain (CTD) alone. Curve represents the average of n=3 replicates.

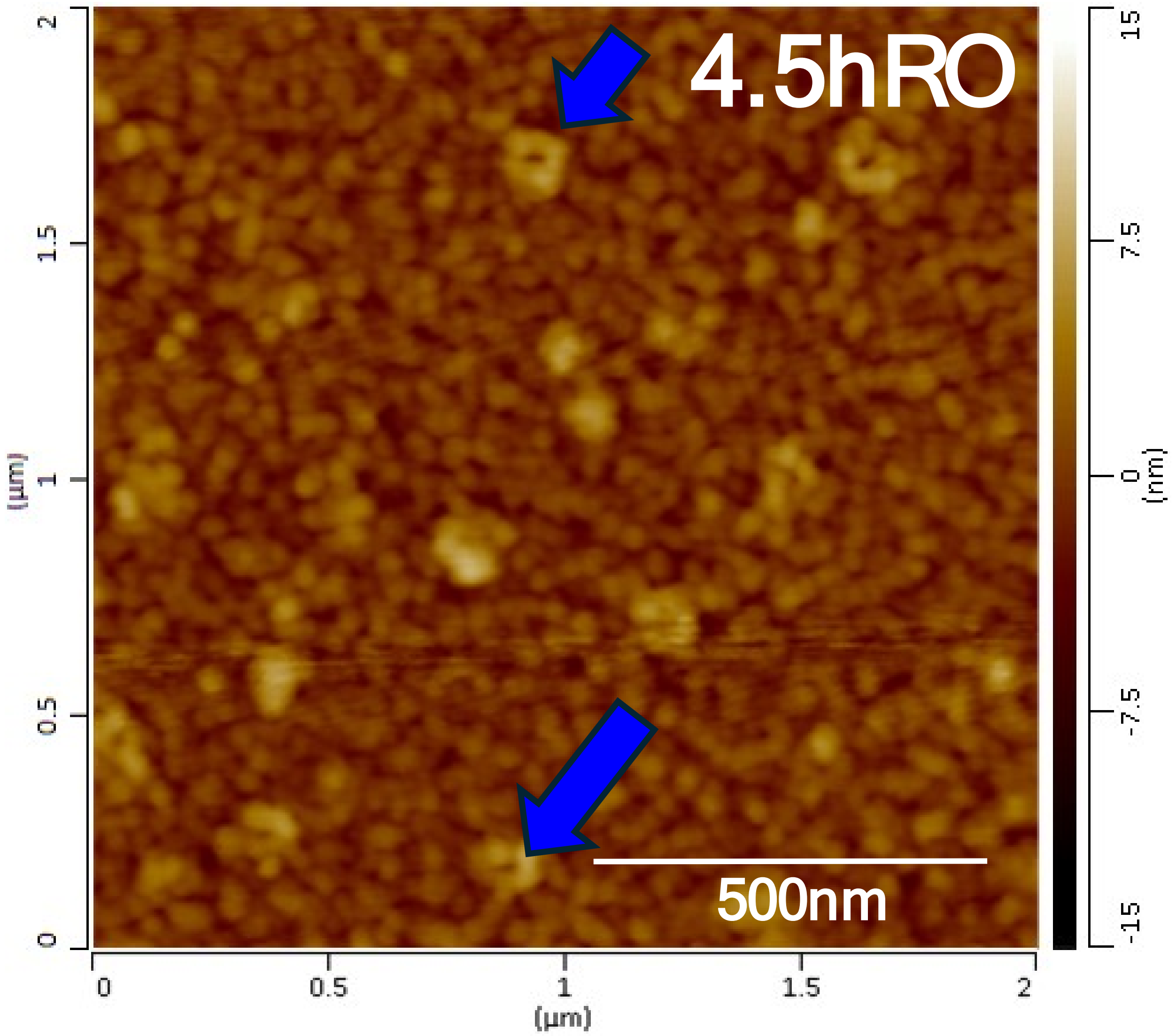

Figure S2: AFM image of CTD TDP-43 DO observed at the late stages of protein aggregation.
